# On the Proper Treatment of the N400 and P600 in Language Comprehension

**DOI:** 10.3389/fpsyg.2017.01327

**Published:** 2017-08-02

**Authors:** Harm Brouwer, Matthew W. Crocker

**Affiliations:** Department of Language Science and Technology, Saarland University Saarbrücken, Germany

**Keywords:** N400, P600, event-related potentials (ERPs), language comphrension, component overlap, task dependence

Event-Related Potentials (ERPs)—stimulus-locked, scalp-recorded voltage fluctuations caused by post-synaptic neural activity—have proven invaluable to the study of language comprehension. Of interest in the ERP signal are systematic, reoccurring voltage fluctuations called *components*, which are taken to reflect the neural activity underlying specific computational operations carried out in given neuroanatomical networks (cf. Näätänen and Picton, 1987). For language processing, the N400 component and the P600 component are of particular salience (see Kutas et al., 2006, for a review). The typical approach to determining whether a target word in a sentence leads to differential modulation of these components, relative to a control word, is to look for effects on mean amplitude in predetermined time-windows on the respective ERP waveforms, e.g., 350–550 ms for the N400 component and 600–900 ms for the P600 component. The common mode of operation in psycholinguistics, then, is to tabulate the presence/absence of N400- and/or P600-effects across studies, and to use this categorical data to inform neurocognitive models that attribute specific functional roles to the N400 and P600 component (see Kuperberg, 2007; Bornkessel-Schlesewsky and Schlesewsky, 2008; Brouwer et al., 2012, for reviews).

Here, we assert that this Waveform-based Component Structure (WCS) approach to ERPs leads to inconsistent data patterns, and hence, misinforms neurocognitive models of the electrophysiology of language processing. The reason for this is that the WCS approach ignores the *latent component structure* underlying ERP waveforms (cf. Luck, 2005), thereby leading to conclusions about component structure that do not factor in *spatiotemporal component overlap* of the N400 and the P600. This becomes particularly problematic when spatiotemporal component overlap interacts with differential P600 modulations due to task demands (cf. Kolk et al., 2003). While the problem of spatiotemporal component overlap is generally acknowledged, and occasionally invoked to account for within-study inconsistencies in the data, its implications are often overlooked in psycholinguistic theorizing that aims to integrate findings across studies. We believe WCS-centric theorizing to be the single largest reason for the lack of convergence regarding the processes underlying the N400 and the P600, thereby seriously hindering the advancement of neurocognitive theories and models of language processing.

## Why the data are inconsistent

**Figure 1:**
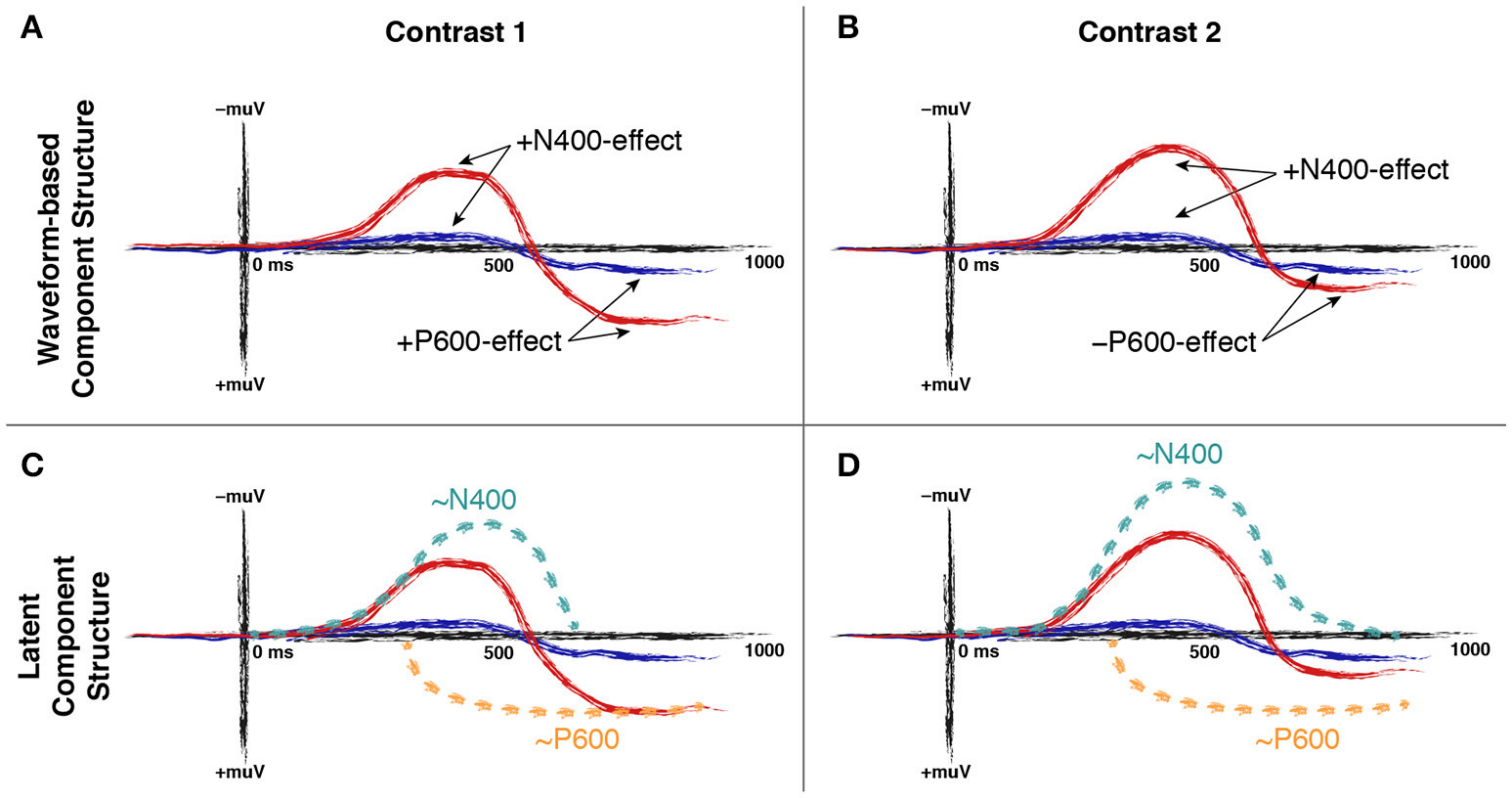
Hypothesized waveforms for two contrasts. The top panels **(A,B)** show the WCS for these contrasts, and the bottom panels **(C,D)** the hypothesized LCS underlying these waveforms. In **(A–D)**, the red line depicts the waveform observed for a target condition, and the blue line the waveform observed for a control condition. In **(C,D)**, the target condition (red line) is decomposed into a latent N400 component (dashed green line) and a latent P600 component (dashed yellow line). See text for details.

ERP studies examining the processing of semantic incongruity sometimes report contradictory results. To shed light on these contradictions, Van Petten and Luka (2012) (henceforth VP&L) conducted a systematic review on semantic incongruity effects. VP&L selected studies comparing incongruent to congruent sentence-final words—e.g., “He spread the warm bread with *socks*/*butter*” (Kutas and Hillyard, 1980)—in healthy adults, using sentences that were otherwise syntactically felicitous, and procedures that did not have an explicit by-item acceptability or judgment task. As these studies were mostly targeted at the N400 component, statistics for the P600 time-window were not always available; if they were not, a P600-effect was judged to be present if the difference in this time-window was at least half as large as the effect in the preceding N400 time-window. VP&L's literature search yielded 45 studies with a total of 64 incongruent/congruent contrasts. They observed that 21 (≈33%) contrasts produced a biphasic N400/P600-effect (cf. Figure 1A), whereas 43 (≈67%) contrasts produced an N400-effect only (cf. Figure 1B).

Hence, on the WCS approach, one third of a set of contrasts—selected to be as homogeneous as possible—produces a biphasic N400/P600-pattern, whereas the other two thirds produce an N400-effect only. Crucially, as VP&L pointed out, this set of results is internally inconsistent. At most, one would predict quantitative differences between contrasts, not qualitative differences. Any viable theory or model of the N400 and the P600 in language comprehension must address this apparent inconsistency in the elicitation pattern of the P600—regardless of whether it predicts a P600-effect to be present or absent for semantic incongruities—in order to successfully capture the full data spectrum.

## Waveform-based vs. latent component structure

The WCS approach to ERPs derives component structure—e.g., the modulation pattern of the N400 and P600 component—from the observable waveform by looking at effects on mean amplitude in predetermined time-windows (but see Groppe et al., 2011a,b, for an alternative approach). The (implicit) logic behind this is that peak amplitudes (maxima or minima in voltages) in these time-windows are indicative of the components of interest. However, this logic violates an important principle of ERP interpretation: A peak is *not* the same thing as a component, and the point at which an ERP waveform peaks carries no significance in itself (cf. Luck, 2005). At any given point, a waveform merely shows the summation of the *latent* components contributing to the ERP signal at that time. Indeed, the processes underlying different components may temporally overlap, and the ERP signal at any given point may be composed of multiple components. Moreover, at different electrode sites, the precise composition of these components may vary, depending on the location of their generators. Hence, at any electrode, peaks in the observed waveform are nothing more than epiphenomena of the underlying Latent Component Structure (LCS)—the set of components contributing to the scalp-recorded ERP signal—and due to *spatiotemporal component overlap*, this LCS may look very different from the WCS.

We can now distinguish between WCS-derived N400- and P600-*effects*, and the N400 and the P600 as *latent components*. Consider the idealized waveforms depicted in Figure 1. The top row shows the WCS for two contrasts between a target (red line) and a control condition (blue line), one contrast producing a biphasic N400/P600-effect (Figure 1A), and one producing an N400-effect only (Figure 1B). The bottom row, in turn, decomposes the target condition (red line) of each of these contrasts into a hypothesized LCS underlying its waveform. On this LCS perspective, both contrasts *do* lead to an increase in the amplitude of the latent P600 component (dashed yellow line), and modulate it to an equal degree. Crucially, the contrasts differ in the degree of modulation of the latent N400 component (dashed green line). Spatiotemporal overlap between the N400 and the P600 causes the larger N400 (contrast 2) to obscure the P600 in the WCS, whereas the smaller N400 (contrast 1) does not. Hence, when the latent N400 and P600 are both modulated to a strong enough extent, LCS and WCS may be in qualitative agreement on component structure (contrast 1). If, on the other hand, one latent component is modulated to a greater degree than the other, WCS and LCS may qualitatively disagree (contrast 2).

We now consider possible LCS-derived explanations for the apparent WCS-derived inconsistencies in the data. More specifically, given the robustness of the N400-effect across the studies reviewed by VP&L, there are effectively two LCS accounts to consider. The first entails an explanation in which spatiotemporal overlap between the latent N400 and the latent P600 in the control condition leads to the spurious *presence* of a P600-effect for the target condition relative to control, that is, a constellation in which the latent P600 for the control condition is less positive than the latent P600 in the target condition. However, as the control condition is always less negative in the N400 time-window, there is no plausible way for the latent P600 to be manifest as less positive than the target in the WCS. The second account, in turn, entails an explanation in which the *absence* of a P600-effect is due to spatiotemporal overlap between the latent N400 and the latent P600. For this account, there are two interacting factors that could explain why the P600 does not survive overlap with the N400. Firstly, target items may simply vary in degree of N400 modulation, and hence in the degree of P600 attenuation. This predicts that if the incongruent sentence-final words get replaced by congruent, but unexpected (non-zero Cloze) completions, N400 amplitudes for target items should be attenuated, rendering the P600 more likely to survive spatiotemporal overlap and be visible in the WCS. Consistent with this prediction, VP&L report 13 studies with a total of 27 unexpected/expected contrasts, 18 (≈66%) of which indeed produce a P600-effect^1^. Secondly, as we will argue below, the absence of a by-item task in the studies reviewed by VP&L induces an overall bias toward attenuated P600 modulations.

## Task-dependence of the P600

It is well-established that the processes underlying the P600 are strongly task-dependent; that is, if an experiment does not involve a by-item acceptability or plausibility judgment task, this typically leads to attenuation of P600 amplitude (e.g., Kolk et al., 2003 and Schacht et al., 2014; see Kuperberg, 2007 and Brouwer et al., 2012 for discussion). Crucially, VP&L specifically selected the studies included in their review to not have an overt by-item task, thereby skewing the distribution of findings toward attenuation of the P600. The significance of this becomes particularly apparent when one considers the interaction between task-dependence and spatiotemporal component overlap: Task-driven attenuation of the P600 is predicted to have a more pronounced effect on WCS when a target condition increases both N400 and P600 amplitude relative to control, compared to when it only increases P600 amplitude.

Two experiments by Kolk et al. (2003) support this prediction. In a first experiment with an explicit acceptability judgment task, they found that a semantically incongruous contrast produced a biphasic N400/P600-effect relative to control, and that a semantic reversal anomaly—e.g., a sentence describing a fox hunting poachers—produced a P600-effect only. In a second experiment, the judgment task was removed, and whereas the P600-effect to the reversal anomaly persisted, the semantic incongruity now produced an N400-effect only. This logic can be applied in reverse to the studies reviewed by VP&L: If these studies were repeated with a by-item acceptability judgment task, the P600 is predicted to survive spatiotemporal overlap with the N400, and be apparent for all semantic incongruities and unexpectancies in the WCS.

## Investigating latent component structure

To the extent that the goal of ERP-based investigations of language is to inform our understanding of the underlying computational operations involved, researchers should be concerned with LCS rather than WCS. While the importance of LCS is generally acknowledged, psycholinguistic theorizing is predominantly WCS-centric (but see Hagoort, 2003, for a notable exception), thereby lagging behind other fields in which the implications of LCS have long been incorporated in interpreting ERP findings (see Squires et al., 1975; Donchin et al., 1978; Näätänen, 1982, among others). Hence, psycholinguistics should go beyond just acknowledging the problem, and start factoring in the implications of LCS in integrating findings within and across studies. Ideally, this should also lead to a shift from studying WCS to studying LCS. However, as the scalp-recorded ERP signal is a summation of latent components, it obscures their independent contributions, and hence investigating the LCS underlying this signal is extremely challenging. Nonetheless, experimental design considerations as well as the (complementary) use of different analysis techniques may help to mitigate this challenge.

First of all, experimental designs could incorporate contrasts that focus on isolating a single component of the signal by keeping effects of spatiotemporal overlap constant between conditions. For instance, if the component of interest is the N400, spatiotemporal overlap with the P600 could be reduced as much as possible by running the experiment without any explicit task. If, on the other hand, the P600 is the component of interest, a relevant task should be used, as well as designs that keep the degree of N400 modulation constant, for instance by using context manipulation designs in which the target word is primed to an equal degree across conditions (cf. reversal anomalies; see Brouwer et al., 2012, for discussion).

Secondly, analysis techniques could be explored that go beyond identifying ERP components by applying ANOVAs or LMEMs on pre-determined time-windows. Such techniques include methods that decompose the signal into principal (PCA; Donchin and Heffley, 1978) or independent components (ICA; Makeig et al., 1997), those that aim to identify processing stages from the signal (using HSMMs; Borst and Anderson, 2015; Anderson et al., 2016), mass univariate analysis (Groppe et al., 2011a,b), regression-based estimation of the waveform (rERPs; Smith and Kutas, 2015a,b), and time-frequency analysis (TFA; Pfurtscheller and Da Silva, 1999; Roach and Mathalon, 2008). These methods cannot solve the problem of the obscured signal itself, but may offer complementary insights that help unravel the underlying LCS. For instance, Regel et al. (2014) show that P600 activity yields a specific frequency profile (power increase/decrease) as determined by TFA. Hence, for a given signal, the presence of such a frequency profile (in the average time-frequency representation) might be indicative of the presence of a P600, even though no P600-effect is present in the WCS.

Finally, LCS could be investigated through bottom-up modeling of the ERP signal. Data from complementary neuroimaging methods (e.g., fMRI and PET) and lesion studies, as well as neuroanatomical models of language electrophysiology (e.g., Brouwer and Hoeks, 2013) could be used to constrain and guide source modeling (see Elting et al., 2003, for a decomposition of the P300 into the P3a and P3b). Moreover, temporal overlap of the N400 and P600 could be modeled within explicit neurocomputational models of ERPs (Alday et al., 2014; Brouwer et al., 2017). Brouwer et al. (2017), for instance, show that their neurocomputational model of the N400 and the P600 best accounts for ERP data on semantic processing if spatiotemporal overlap between these components is taken into account.

## Conclusion

Event-Related Potentials (ERPs) are invaluable to the study of language comprehension, but psycholinguistics often gets them wrong. The standard mode of operation is to tabulate the presence/absence of effects on mean amplitude in predetermined time-windows, which are then taken to be indicative of language-sensitive ERP components, such as the N400 and the P600. The VP&L review on the processing of semantic incongruity shows that this WCS approach leads to inconsistent data patterns. We have argued that when faced with such apparent inconsistencies in patterns of WCS-derived N400- and/or P600-effects across studies with similar manipulations, the LCS underlying the observed waveforms may offer a principled explanation for the observed variance. The observed WCS may (minimally) be decomposed into the contribution of latent N400 and P600 components, which may overlap spatiotemporally in the ERP signal. Importantly, these latent components are known to be attenuated and/or amplified in systematic ways: For instance, N400 amplitude is sensitive to the degree of semantic expectancy, while P600 amplitude is modulated by the nature of the comprehension task. It is the interplay between the systematic modulation of these latent components, due to their spatiotemporal overlap in the observed ERP signal, which explains the variance in WCS-derived effects, such as those observed for the studies reviewed by VP&L. We believe that in order to arrive at a viable neurocognitive model of language processing, it is essential to incorporate the implications of LCS into psycholinguistic theorizing.

## Author contributions

All authors listed have made a substantial, direct and intellectual contribution to the work, and approved it for publication.

### Conflict of interest statement

The authors declare that the research was conducted in the absence of any commercial or financial relationships that could be construed as a potential conflict of interest.

## Footnotes

^1^While the P600-effect for incongruent/congruent contrasts was predominantly distributed parietally, the unexpected/expected contrasts produced a more frontal distribution.

## References

[B1] Alday P. M. Schlesewsky M. Bornkessel-Schlesewsky I. (2014). Towards a computational model of actor-based language comprehension. Neuroinformatics 12, 143–179. 10.1007/s12021-013-9198-x 23912508

[B2] Anderson J. R. Zhang Q. Borst J. P. Walsh M. M. (2016). The discovery of processing stages: Extension of Sternberg's method. Psychol. Rev. 123:481 10.1037/rev0000030 PMC5033670 27135600

[B3] Bornkessel-Schlesewsky I. Schlesewsky M. (2008). An alternative perspective on “Semantic P600” effects in language comprehension. Brain Res. Rev. 59, 55–73. 10.1016/j.brainresrev.2008.05.003 18617270

[B4] Borst J. P. Anderson J. R. (2015). The discovery of processing stages: analyzing eeg data with hidden semi-markov models. NeuroImage 108, 60–73. 10.1016/j.neuroimage.2014.12.029 25534112

[B5] Brouwer H. Crocker M. W. Venhuizen N. J. Hoeks J. C. (2017). A neurocomputational model of the N400 and the P600 in language processing. Cogn. Sci. 41, 1318–1352. 10.1111/cogs.12461 28000963 PMC5484319

[B6] Brouwer H. Fitz H. Hoeks J. (2012). Getting real about semantic illusions: rethinking the functional role of the P600 in language comprehension. Brain Res. 1446, 127–143. 10.1016/j.brainres.2012.01.055 22361114

[B7] Brouwer H. Hoeks J. C. (2013). A time and place for language comprehension: mapping the N400 and the P600 to a minimal cortical network. Front. Hum. Neurosci. 7:758. 10.3389/fnhum.2013.00758 24273505 PMC3824103

[B8] Donchin E. Heffley E. F. III. (1978). Multivariate analysis of event-related potential data: a tutorial review, in Multidisciplinary Perspectives in Event- Related Brain Potential Research, ed Otto D. (Washington, DC: U.S. Government Printing Office), 555–572.

[B9] Donchin E. Ritter W. McCallum W. (1978). Cognitive psychophysiology: the endogenous components of the ERP, in Event-Related Brain Potentials in Man, eds Callaway E. Tueting P. Koslow S. H. (New York, NY: Academic Press), 349–411.

[B10] Elting J. W. van Weerden T. van der Naalt J. De Keyser J. Maurits N. (2003). P300 component identification using source analysis techniques: reduced latency variability. J. Clin. Neurophysiol. 20, 26–34. 10.1097/00004691-200302000-00003 12684555

[B11] Groppe D. M. Urbach T. P. Kutas M. (2011a). Mass univariate analysis of event-related brain potentials/fields I: a critical tutorial review. Psychophysiology 48, 1711–1725. 10.1111/j.1469-8986.2011.01273.x 21895683 PMC4060794

[B12] Groppe D. M. Urbach T. P. Kutas M. (2011b). Mass univariate analysis of event-related brain potentials/fields II: simulation studies. Psychophysiology 48, 1726–1737. 10.1111/j.1469-8986.2011.01272.x 21895684 PMC4059014

[B13] Hagoort P. (2003). Interplay between syntax and semantics during sentence comprehension: ERP effects of combining syntactic and semantic violations. J. Cogn. Neurosci. 15, 883–899. 10.1162/089892903322370807 14511541

[B14] Kolk H. H. Chwilla D. J. Van Herten M. Oor P. J. (2003). Structure and limited capacity in verbal working memory: a study with event-related potentials. Brain Lang. 85, 1–36. 10.1016/S0093-934X(02)00548-5 12681346

[B15] Kuperberg G. R. (2007). Neural mechanisms of language comprehension: challenges to syntax. Brain Res. 1146, 23–49. 10.1016/j.brainres.2006.12.063 17400197

[B16] Kutas M. Hillyard S. A. (1980). Reading senseless sentences: brain potentials reflect semantic incongruity. Science 207, 203–205. 10.1126/science.7350657 7350657

[B17] Kutas M. van Petten C. Kluender R. (2006). Psycholinguistics electrified II: 1994–2005, in Handbook of Psycholinguistics, 2nd Edn. eds Traxler M. J. Gernsbacher M. A. (New York, NY: Elsevier), 659–724.

[B18] Luck S. J. (2005). An introduction to the Event-Related Potential Technique. Cambridge, MA: MIT Press.

[B19] Makeig S. Jung T.-P. Bell A. J. Ghahremani D. Sejnowski T. J. (1997). Blind separation of auditory event-related brain responses into independent components. Proc. Natl. Acad. Sci. U.S.A. 94, 10979–10984. 10.1073/pnas.94.20.10979 9380745 PMC23551

[B20] Näätänen R. (1982). Processing negativity: an evoked-potential reflection. Psychol. Bull. 92:605 10.1037/0033-2909.92.3.605 7156260

[B21] Näätänen R. Picton T. (1987). The N1 wave of the human electric and magnetic response to sound: a review and an analysis of the component structure. Psychophysiology 24, 375–425. 10.1111/j.1469-8986.1987.tb00311.x 3615753

[B22] Pfurtscheller G. Da Silva F. L. (1999). Event-related eeg/meg synchronization and desynchronization: basic principles. Clin. Neurophysiol. 110, 1842–1857. 10.1016/S1388-2457(99)00141-8 10576479

[B23] Regel S. Meyer L. Gunter T. C. (2014). Distinguishing neurocognitive processes reflected by P600 effects: Evidence from ERPs and neural oscillations. PLoS ONE 9:e96840. 10.1371/journal.pone.0096840 24844290 PMC4028180

[B24] Roach B. J. Mathalon D. H. (2008). Event-related eeg time-frequency analysis: an overview of measures and an analysis of early gamma band phase locking in schizophrenia. Schizophr. Bull. 34, 907–926. 10.1093/schbul/sbn093 18684772 PMC2632478

[B25] Schacht A. Sommer W. Shmuilovich O. Martíenz P. C. Martín-Loeches M. (2014). Differential task effects on N400 and P600 elicited by semantic and syntactic violations. PLoS ONE 9:e91226. 10.1371/journal.pone.0091226 24614675 PMC3948820

[B26] Smith N. J. Kutas M. (2015a). Regression-based estimation of erp waveforms: I. the rerp framework. Psychophysiology 52, 157–168. 10.1111/psyp.12317 25141770 PMC5308234

[B27] Smith N. J. Kutas M. (2015b). Regression-based estimation of erp waveforms: II. nonlinear effects, overlap correction, and practical considerations. Psychophysiology 52, 169–181. 10.1111/psyp.12320 25195691 PMC5306445

[B28] Squires N. K. Squires K. C. Hillyard S. A. (1975). Two varieties of long-latency positive waves evoked by unpredictable auditory stimuli in man. Electroencephalogr. Clin. Neurophysiol. 38, 387–401. 10.1016/0013-4694(75)90263-1 46819

[B29] Van Petten C. Luka B. J. (2012). Prediction during language comprehension: benefits, costs, and ERP components. Int. J. Psychophysiol. 83, 176–190. 10.1016/j.ijpsycho.2011.09.015 22019481

